# Differential roles of hippocampal glutamatergic receptors in neuropathic anxiety-like behavior after partial sciatic nerve ligation in rats

**DOI:** 10.1186/s12868-015-0150-x

**Published:** 2015-03-14

**Authors:** Xue-Qin Wang, Xiao-Lin Zhong, Zhi-Bin Li, Hong-Tao Wang, Juan Zhang, Fang Li, Jian-Yi Zhang, Ru-Ping Dai, Zhou Xin-Fu, Chang-Qi Li, Zhi-Yuan Li, Fang-Fang Bi

**Affiliations:** Center for Experimental Medicine, the Third Xiangya Hospital, Central South University, Tongzipo Road 138, Changsha, Hunan China; Department of Anesthesiology, the Third Xiangya Hospital, Central South University, Tongzipo Road 138, Changsha, Hunan China; Department of Anatomy and Neurobiology, School of Basic Medical Science, Central South University, Tongzipo Road 172, Changsha, Hunan China; Department of Neurology, XiangYa Hospital, Central South University, XiangYa Road 88, Changsha, Hunan China; Department of Anesthesia, the Second XiangYa Hospital of Central South University, Ren-Min Road 86, Changsha, Hunan China; School of Pharmacy and Medical Sciences, Sansom Institute, University of South Australia, Adelaide, SA 5000 Australia

**Keywords:** Neuropathic pain, Hippocampus, Glutamatergic receptor, Anxiety, Rats

## Abstract

**Background:**

Neuropathic pain evoked by nerve injury is frequently accompanied by deterioration of emotional behaviors, but the underlying signaling mechanisms remain elusive. Glutamate (Glu) is the major mediator of excitatory synaptic transmission throughout the brain, and abnormal activity of the glutamatergic system has been implicated in the pathophysiology of pain and associated emotional comorbidities. In this study we used the partial sciatic nerve ligation (PSNL) model of neuropathic pain in rats to characterize the development of anxiety-like behavior, the expression of glutamatergic receptors, and the phosphorylation of extracellular signal-regulated kinase (ERK) in the hippocampus, the region that encodes memories related to emotions.

**Results:**

We found that the mechanical withdrawal threshold was significantly reduced and an anxiety-like behavior was increased as determined via open field tests and elevated plus-maze tests at 28 days after injury. No significant differences were found in the ratio of sucrose preference and immobility time detected by sucrose preference tests and forced swimming tests respectively, possibly due to the timing factor. The expression of N-methyl-D-aspartate (NMDA) receptor subtypes NR1 and NR2B, but not NR2A, GluR1, or GluR2 (the main subtype of the α-amino-3-hydroxy-5-methyl-4-isoxazolepropionic acid [AMPA] receptor) in the hippocampus of injured rats was significantly reduced. Moreover, PSNL resulted in decreased phosphorylation of ERK1/2 in the hippocampus. Intriguingly, treatment with D-serine (a co-agonist of NMDA receptor, 1 g/kg intraperitoneally) reduced the anxiety-like behavior but not the mechanical hypersensitivity induced by PSNL.

**Conclusions:**

PSNL can induce significant anxiety-like but not depression-like behavior, and trigger down-regulation of NMDA but not AMPA receptors in the hippocampus at 28 days after injury.

## Background

Pain is related to sensory and affective parameters and accompanied by feelings of unpleasantness [1,2]. Chronic neuropathic pain arising from a lesion or dysfunction within the nervous system is a prevalent clinical problem and extremely difficult to treat because the perception of pain is associated with conditions such as anxiety and depression [3,4]. Numerous studies have shown that pain-related mood disorders affect the perception of pain and reduce the pain threshold [5,6]. However, the underlying mechanisms of pain- associated behaviors that arise from the brain are poorly understood.

To date, human and animal studies have indicated that supraspinal structures are involved in pain. In particular, the hippocampus as a major part of the limbic system has been the subject of research interest in recent years because of its relation to the emotional component of pain [7,8]. For example, one study found that emotional abnormalities associated with chronic back pain are related to the reorganization of processing within the hippocampus, and between the hippocampus and the cortex [9]. Another study reported that the synaptic plasticity in the hippocampus is involved in the neuropathic pain condition based on the observation that hippocampal long-term potentiation is impaired after peripheral nerve injury [10]. The hippocampus also plays a vital regulatory role in mood and depressive symptoms in burning mouth syndrome, one form of chronic pain, with increased gray matter volume (GMV) in the hippocampus and increased connectivity between bilateral hippocampus/amygdala and medial prefrontal cortex [11]. The optimal management of neuropathic pain including the alleviation of anxiety and molecular mechanisms underlying the supraspinal structures have been studied in animal models of nerve injury [12,13]. Nevertheless, the changes in cellular regulatory and signaling processes within the hippocampus in neuropathic pain induced by partial sciatic nerve ligation (PSNL) have remained minimally explored.

Nerve injury affects synaptic plasticity and induces changes in multiple neurotransmitters and the transmission of intracellular signaling, through which the expression or function of excitatory and inhibitory receptors is altered [14,15]. Glutamate is a major excitatory neurotransmitter of the central nervous system, and recent studies have investigated the potential roles of the glutamatergic system in the pathophysiology and treatment of mood disorders [16,17]. Expression of NMDA receptors (NMDARs) including the NR1, NR2A and NR2B subunits in spinal dorsal root ganglion and primary afferents is believed to be an important mechanism underlying chronic neuropathic pain [18,19]. Mild noxious heat stimuli under a neuropathic pain condition induced by a peripheral nerve injury may increase the release of glutamate and the number of phosphorylated-NR1-positive cells in the cingulate cortex [20].

The objective of this study was to elucidate the cellular and molecular mechanisms responsible for the perception of pain and the development of neuropathic pain–induced behavior by measuring the expression of hippocampal glutamatergic receptors following a peripheral neuropathic injury that induces a chronic pain state mimicking the clinical neuropathic pain induced by nerve injury.

## Methods

### Animals

Male Sprague–Dawley rats from Central South University Animal Services (Changsha, China) weighing 220–250 g were used. The experimental protocol was approved by the Animal Care and Use Committee of Central South University and conformed to the National Institutes of Health Guide for the Care and Use of Laboratory Animals. All efforts were made to minimize the number of rats used and their suffering. Animals were housed under controlled temperature (23 ± 2°C), relative humidity (50 ± 10%), and artificial lighting (lights on from 7 am until 7 pm) with distilled water and food available ad libitum. A right partial sciatic nerve ligation (PSNL) model was established according to the method of Seltzer et al. [21] with some modifications. Under anesthesia (injection by administration of a dose of 10% chloral hydrate, 40 mg/kg,intraperitoneal), approximately one half of the nerve was ligated with an absorbable 6–0 braided silk suture tied loosely around the right sciatic nerve at the middle point of the long axis of the thigh. For the control group, the rats were subjected to the same procedure without nerve ligation. Different rats were used for different procedures so that no animal experienced more than one session, and the rats were randomly assigned to procedures as described below.

### Nociceptive testing

To validate the neuropathic pain model, we used the mechanical withdrawal threshold (MWT), a classic test for mechanical allodynia and the most reliable parameter to assess neuropathic pain after nerve injury [22,23]. Mechanical allodynia was assayed using nylon von Frey filaments according to the “up-down” algorithm as described in our previous studies [24,25]. Nociceptive testing was performed 2 days before surgery to obtain baseline data. Mechanical allodynia was determined at 7, 14, 21 and 28 days after PSNL (n = 8/ group). The testing was performed by two investigators who were blinded to the experimental treatment. Rats were placed on wire mesh platforms in clear cylindrical plastic enclosures, and after 20 minutes of acclimation, filaments were applied to the center of the plantar surface of the hind paw and left in place for 5 s. Withdrawal of the hind paw from the floor was scored as a response. When no response was obtained, the next stiffer filament in the series was applied to the same paw, if a response was obtained, a less stiff filament was applied further. Testing proceeded in this manner until four fibers had been applied after the first one to cause a withdrawal response, allowing estimation of the mechanical withdrawal threshold.

### Elevated plus-maze test

Anxiety-like behavior was evaluated using the elevated plus-maze (EPM) test as described in our previous studies at 28 days after PSNL [24-27]. Briefly, after habituation for approximately 1 day in the experimental room, rats were placed in the middle of the EPM apparatus, and their behavior was recorded by a video camera for 5 min. Data were expressed as the percentage of time spent in the open-arms as an index of anxiety-like behavior. The number of open arm and closed-arm entries was calculated during the testing session. The maze was cleaned after each trial.

### Open field test

The open field test (OFT) was applied as described previously to assess anxiety-like behavior in the experimental animals at 28 day after PSNL [24-27]. Briefly, animals were placed directly into the center of the open field (100 × 100 × 48 cm, length × width × height) which was divided equally into 25 squares. Movement of each animal in the area was recorded during the 5-min testing session. An observer blinded to the experimental conditions coded the videotapes. The time spent in the center of the open field were calculated. A greater amount of time spent in the center of the nine squares indicated a lower level of anxiety. Overall locomotor activity was defined as the total number of squares crossed during the testing session.

### Forced swimming test

The forced swimming test (FST) is used often as an animal model for assessing depression-like behavior. The experiments in this study were carried out as described in our previous study at 28 days after PSNL [28]. Briefly, the rats were placed individually in a glass cylinder (40 × 18 cm, height × diameter) containing water up to 30 cm at 23°C. After 15 min, they were returned to a 30°C drying environment (the pre-test). Twenty-four hours later, the rats were returned to the cylinder for 5 min (the test), and this session was recorded with a video camera. Experiments were performed between 14:00 and 16:00 pm. An experimenter, who was blinded to the animal treatments, observed the videotapes. The total immobility time was recorded, and a rat was considered immobile when floating and making only the necessary movements to keep its head above the water surface.

### Sucrose preference test

The sucrose preference test (SPT), a two-bottle choice paradigm, was carried out according to the procedure used in our previous study to estimate the depression-like behavior at 28 days after PSNL [28]. Briefly, the rats were free to choose a 2% sucrose solution or water for 2 consecutive days, and the positions of two bottles were altered once during the test. The consumption of water and sucrose solution was measured by volume. The preference for 2% sucrose was calculated from the average solution consumption in the 2 days and expressed as a percentage of the total amount of liquid consumed.

### Immunohistochemistry

At 28 days after PSNL, one set of rats (n = 4 or 6 for each group) was sacrificed by an overdose of chloral hydrate (80 mg/kg). Immunohistochemical staining for GluR1, GluR2, NR1, NR2A, NR2B, and p-ERK1/2 was performed as described in previous studies [15,29-31] with a slight modification. Briefly, brains were fixed for 4 h in 4% paraformaldehyde after perfusion and cryoprotection by immersion in 20% sucrose in phosphate buffer (pH = 7.4) overnight. Transverse sections of the brain (30 μm) were cut using a cryostat and mounted on 3-aminopropyl triethoxy- silane- coated slides. As described in our prior studies but with some modifications [31], 15 slices of hippocampus were randomly obtained from eight sets of serial slices from each rat at – 2.3 mm to – 5.8 mm anteroposterior to the bregma for immunostaining. Rabbit anti-NR1 (1:1000; Epitomics, USA, catalog number: 8173–1), NR2A (1:1000; Epitomics, USA, catalog number: 3916–1), NR2B (1:1000; Abcam, USA, catalog number: ab81271), GluR1 (1:500; Epitomics, USA, catalog number: 3861–1), GluR2 (1:1000; Epitomics, USA, catalog number: 3520–1), and p-ERK1/2 (1:1000; Cell Signaling Technology, USA, catalog number: 4695) were incubated at 4°C overnight. The sections were washed with 0.1% Triton X-100 solution in 0.1 M phosphate-buffered saline (PBS) and further incubated with the VECTASTAIN ABC Kit (Vector Laboratories, USA, catalog number: PK- 4001), which contained biotinylated goat anti-rabbit IgG secondary antibody (1:500) and concentrated A and B reagents. Diaminobenzidine tetrahydrochloride (DAB, Sigma, USA, 1:2000, catalog number: D5905) was used as a peroxidase substrate. The average optic density (OD) of positive staining was detected in regions of CA1, CA2 and CA3 at the same magnification factor and light intensity by an author who was blinded to treatments using HPIAS-1000 image analysis as described in previous studies [25,31,32]. Data were acquired for 15 sections/brain region/animal and averaged to produce a single value per animal.

### Western blotting

Semi-quantified Western blotting was used to assess the expression of GluR1, GluR2, NR1, NR2A and NR2B in the ipsilateral hippocampus and performed as described for our recent study [27,33] with a slight modification. Another set of rats (n = 8 for each group) was sacrificed under deep anesthesia. The hippocampus was removed and immediately placed on dry ice and stored at −80°C until use. Frozen samples were homogenized in a lysis buffer containing protease inhibitors cocktails (Roche, Germany, catalog number: P8340) and phenylmethanesulfonylfluoride (PMSF, Sigma, USA, catalog number: p7626). Samples were then centrifuged at 10,000 rpm for 15 min at 4°C, and the supernatants were used for Western blotting.

Equal amounts of protein were loaded, separated by 10% Tris-Tricine sodium dodecyl sulfate polyacrylamide gel electrophoresis, and then transferred onto a polyvinilidene difluoride membrane (Amersham Bioscience). After blocking in 10% non-fat milk for 2 h at room temperature, the membrane was incubated overnight at 4°C with rabbit anti-NR1 (1:8000; Epitomics, USA, catalog number: 8173–1), NR2A (1:5000; Epitomics, USA, catalog number: 3916–1), NR2B (1:3000; Abcam, USA, catalog number: ab81271), GluR1 (1:3000; Epitomics, USA, catalog number: 3861–1), GluR2 (1:5000; Epitomics, USA, catalog number: 3520–1), p-ERK1/2 (1:2000; Cell Signaling Technology, USA, catalog number: 4695), and mouse anti-glyceraldehyde-3-phosphate dehydrogenase (GAPDH; 1:2000, Millipore, USA, catalog number: CB1001) primary antibodies. The blots were then incubated with the second antibody, goat anti-rabbit IgG conjugated with horseradish peroxidase (1:2000, Pierce, Rockford, IL, catalog number: AB501-01A) and goat anti-mouse IgG conjugated with horseradish peroxidase (1:2000, Pierce, Rockford, IL, catalog number: AB503-01A) at room temperature for 2 h. Membranes were washed three times with 0.02 M Tris-buffered saline/Tween 20 (TBST) and signals were detected by enhanced chemiluminescence (ECL, ComWin Biotech Co, Beijing, China, catalog number: cw 0048c) followed by exposure to X-ray films. For the quantification of Western blot signals, X- ray films with blotting bands were scanned. The BIORAD Image Analysis System (Bio-Rad Laboratories, Inc., Hercules, CA) was then used to measure the integrated OD of the bands.

### Effects of D-serine on the neuropathic pain-related behavior induced by PSNL

To confirm the role of NMDARs in the anxiety-like behavior, we examined whether treatment with a co-agonist of NMDAR could correct behavioral abnormalities in rats after PSNL. D-serine (Sigma-Aldrich, St. Louis, MO, USA, catalog number: s4250) was dissolved in 0.9% saline. The solutions were prepared immediately prior to the experiment. We injected D-serine (1 g/kg) according to a protocol used in a previous study [34] with some modifications. Animals from the control groups received intraperitoneal injections of saline (vehicle). Rats were subjected to OFT, EPM and nociceptive testing individually 90 min after D-serine or saline treatment.

### Data and statistical analysis

Statistical analysis was performed using Graphpad Prism 5 (Graphpad Software, San Diego, CA). All results are expressed as mean ± standard error (SEM). Differences between groups were compared two-way analysis of variance (ANOVA) followed by Bonferroni testing where appropriate. For Western blot analysis, the protein expression in the control group was normalized to 1, and the relative density of the other groups was calculated proportionately. An unpaired two-tailed Student’s *t* test was used if only two groups were applied. A p value <0.05 was considered to indicate a significant difference.

## Results

### PSNL causes behavioral changes related to neuropathic pain

**Figure 1:**
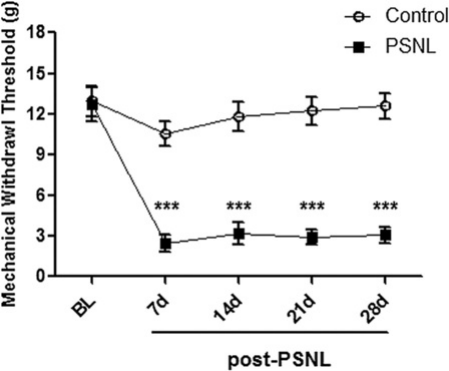
**Changes in MWT over time in rats after partial sciatic nerve ligation.** *** p <0 .001 represents statistically significant differences compared with control group (n = 8/ group). PSNL represents the partial sciatic nerve ligation group. MWT represents mechanical withdrawal threshold.

The persistence of nerve injury-induced hypersensitivity to mechanical stimuli was assessed using nylon von Frey filaments. Consistent with previous findings [21], the MWT was lower 7 days after operation in the PSNL group (2.42 ± 0.62 g) than in the control rats (10.55 ± 0.90 g; n = 8 for each group, p < 0.001, Figure 1). The MWT in PSNL rats remained significantly lower (p < 0.001) than that in control rats at all other time points examined (Figure 1).

### PSNL induces anxiety-like but not depressive-like behavior at 28 days after injury

**Figure 2:**
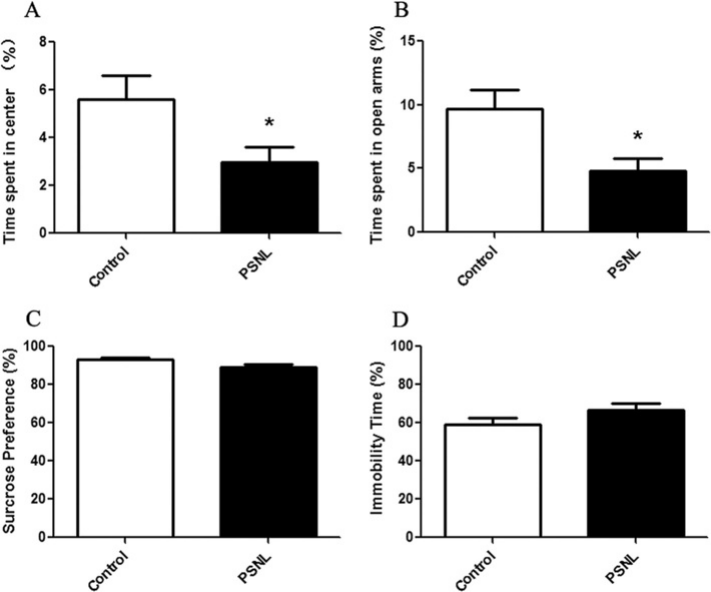
**Development of anxiety-like behavior but not depression-like behavior following PSNL. (A)** Open-field test shows the decrease in the amount of time spent in the central zone after injury (n = 8 for control and n = 9 for PSNL); **(B)** the percentage of time spent in the open arm as detected by the EPM test in the control and PSNL groups (n = 8 for control and n = 7 for PSNL); **(C)** the percentage of preference for sucrose in the sucrose preference test (n = 8 for control and n = 7 for PSNL); **(D)** immobility time in the forced swimming test (n = 7 for control and n = 9 for PSNL), * p < 0.05 represents statistically significant differences compared with control group.

Here, we carried out the OFT and EPM test to assess the anxiety-like behavior in the two groups at 28 days after operation. No significant difference was observed in the total number of squares crossed in the open field test between the PSNL and control rats (106.6 ± 11.33 vs. 99.67 ± 12.58, t = 0.4069, p > 0.05, n = 8 for control and n = 9 for PSNL), although rats in the PSNL group showed tended to exhibit a reduced travel distance compared to the control rats (data not shown). We found that PSNL was effective for inducing anxiety-like behavior. Student’s t-tests indicated that the time spent in the center of the open field was decreased significantly (5.63 ± 0.96% vs. 2.96 ± 0.66%, t = 2.332, p < 0.05, n = 8 for control and n = 9 for PSNL, Figure 2A). In the EPM test, the time spent in the open arm by the PSNL rats was less than that by the control rats (9.68 ± 1.53% vs. 4.81 ± 0.94%, t = 2.610, p < 0.05, n = 8 for control and n = 7 for PSNL, Figure 2B). In addition, there was no difference in the total number of entries in the EPM test between the two groups (8.63 ± 0.73 vs. 9.00 ± 1.02, t = 0.3040, n = 8 for control and n = 7 for PSNL, p > 0.05, data not shown).

Then, we used the SPT to assess depression-like behavior. No difference was observed in the preference for sucrose between the two groups (93.05 ± 1.21% vs. 89.02 ± 1.75%, t = 1.935, p > 0.05, n = 8 for control and n = 7 for PSNL, Figure 2C). We also evaluated the depression-like behavior at 28 days after ligation with the FST. Consistently, there was no difference in the immobility time between the two groups at this time point in the FST (58.90 ± 3.84% vs. 68.56 ± 2.90%, t = 2.047, p > 0.05, n = 7 for control and n = 9 for PSNL, Figure 2D). These results indicate that PSNL did not induce a depression-like behavior at 28 days post operation.

### PSNL alters the expression of NMDA and AMPA receptors in the hippocampus

**Figure 3:**
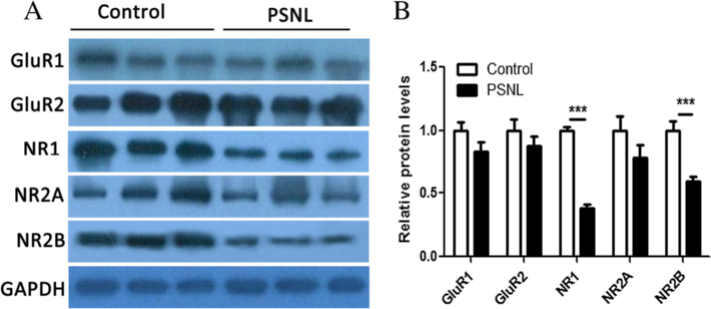
**Effect of partial sciatic nerve ligation on glutamatergic receptors expression in the ipsilateral hippocampus. (A)** GluR1 (MW = 106 kDa), GluR2 (MW = 106 kDa), NR1 (MW = 120 kDa), NR2A (MW = 170 kDa) and NR2B (MW = 180 kDa) expression in the ipsilateral hippocampus using GAPDH (MW = 36 kDa) as the loading control. **(B)** The densitometric analysis for GluR1, GluR2, NR1, NR2A and NR2B. n = 8 for each group, ***p < 0.001 represents statistically significant differences compared with control group. MW represents molecular weight.

**Figure 4:**
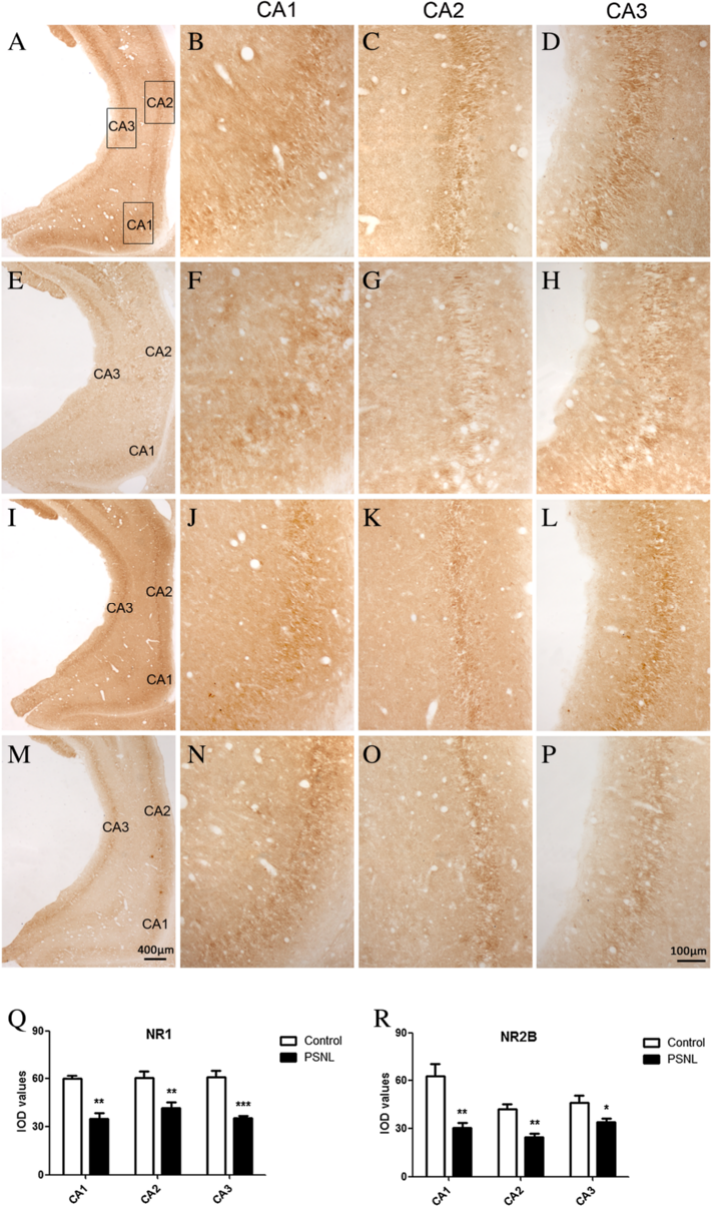
**Effect of partial sciatic nerve ligation on NR1 and NR2B the ipsilateral ventral hippocampus.** Representative coronal sections stained for NR1 **(A** **and** **E)** and NR2B (**I** **and** **M)** in specimens from the control **(A, I)** and PSNL groups **(E, M).** The higher magnification images of NR1 and NR2B staining in CA1, CA2, and CA3 of the ipsilateral hippocampus are displayed for the control **(B, C, D)** and PSNL group **(F, G, H)**; Those for the control **(J, K, L)** and PSNL groups **(N, O, P)** respectively, correspond to the labeled regions in **A**, **E**, **I**, and M. Scale bar: 100 μm in P; 400 μm in M. **(Q** **and** **R)** Quantitative analysis of OD for NR1 and NR2B in ipsilateral sides of CA1, CA2, and CA3 of the whole hippocampus for each group. n = 6 for each group, ***p < 0.001,**p < 0.01,*p < 0.05 represents statistically significant differences compared with control group.

Western blot and immunohistochemistry analysis were conducted to assess the expression of NMDA and AMPA receptors in the hippocampus at 28 days after ligation. The expression of NMDARs and AMPARs in the bilateral hippocampus was significantly altered, We only presented the results for the expression of glutamatergic receptors in the ipsilateral hippocampus. Our results showed that PSNL induced a decrease in NR1 expression (0.385 ± 0.026 vs.1.000 ± 0.029, t = 15.89, p < 0.001, n = 8 for each group, Figure 3) and NR2B (0.591 ± 0.039 vs. 1.000 ± 0.076, t = 4.818, p < 0.001, n = 8 for each group, Figure 3) at the protein level in the ipsilateral hippocampus of PSNL rats relative to expression levels in the control rats. However, no significant difference in the expression of NR2A (0.781 ± 0.108 vs. 1.000 ± 0.113, t = 1.408, n = 8 for each group, p > 0.05), GluR1 (0.833 ± 0.073 vs. 1.000 ± 0.073, t = 1.657, n = 8 for each group, p > 0.05), or GluR2 (0.882 ± 0.068 vs. 1.000 ± 0.091, t = 1.035, n = 8 for each group, p > 0.05) at the protein level was observed between the two groups at postoperative 28 days. Meanwhile, we assessed the expression of NR1 and NR2B in the hippocampus with immunohistochemistry and observed that the expression of NR1 and NR2B was significantly altered in the hippocampus (Figure 4 A-P). Figure 4 shows the positive immunostaining for NR1 (A and E) and NR2B (I and M) in the cytoplasm and process of neurons of the ipsilateral hippocampus of the control (A, I) and PSNL groups (E, M). Student’s *t* test analysis revealed that there were significant differences in the OD for NR1 (Figure 4Q, CA1: 35.04 ± 3.42 vs 60.09 ± 1.66., t = 5.851, p < 0.01; CA2: 41.91 ± 3.41 vs.60.60 ± 4.19, t = 3.459, p < 0.01; CA3: 35.34 ± 1.58 vs. 60.92 ± 4.22, t = 6.222, p < 0.001, n = 6 for each group) and NR2B (Figure 4R, CA1: 30.26 ± 3.24 vs. 62.66 ± 7.66, t = 3.897, p < 0.01; CA2: 24.78 ± 2.07 vs.42.35 ± 2.73, t = 5.133 p < 0.01; CA3: 34.06 ± 2.24 vs. 46.17 ± 4.61, t = 2.364, p < 0.05, n = 6 for each group) and positive staining in the ipsilateral CA1, CA2, and CA3 regions of the hippocampus in the two groups at postoperative 28 days. These results indicate that PSNL can alter the expression of NR1 and NR2B in the hippocampus.

### PSNL decreases phosphorylation of p-ERK1/2 in the hippocampus

**Figure 5:**
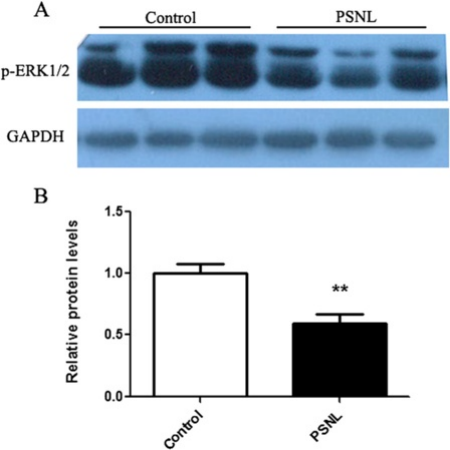
**Effect of partial sciatic nerve ligation on p-ERK1/2 (MW = 44/42 kDa) expression in the ipsilateral hippocampus. (A)** Representative bands are shown for two groups, using GAPDH (MW = 36 kDa) as the loading control; **(B)** densitometric analysis for p-ERK1/2 in the control and PSNL groups. n = 8 for each group, **p < 0.01 represents statistically significant differences compared with control group.

**Figure 6:**
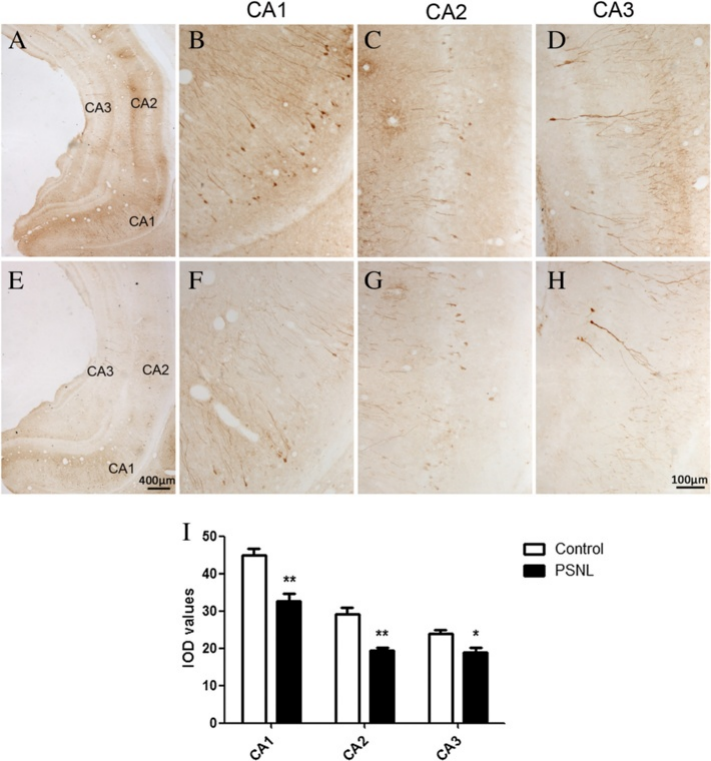
**Representative photomicrographs show p-ERK1/2 immunoreactivity in the CA1, CA2, and CA3 regions of the ipsilateral ventral hippocampus from coronal brain sections between the control (A) and PSNL groups (E). B, C, D** and **F, G, H** are the higher magnification images corresponding to the labeled regions in **A** and **E** respectively. **(I)** Quantitative analysis of the OD of p-ERK1/2 in ipsilateral sides of the CA1, CA2, and CA3 regions of the whole hippocampus for each group. Scale bar: 100 μm in H; 400 μm in **E**. n = 4 for each group, **p < 0.01, *p < 0.05 PSNL vs control.

To understand the mechanisms underlying glutamatergic-mediated pain-related affective behavior, the glutamatergic-dependent signaling pathways must be identified. We postulated that PSNL could affect the phosphorylation of ERK1/2 in the hippocampus. As shown in Figure 5A, Western blot was conducted to confirm the expression of p-ERK1/2 in the ipsilateral hippocampus at 28 days after ligation. The results showed that the level of p-ERK 1/2 was significantly reduced (0.59 ± 0.08 vs.1.00 ± 0.07, t = 3.801, p < 0.01,n = 8 for each group, Figure 5B). There was no significant difference in total ERK1/2 expression at 28 days between the two groups (data not shown). Immunoreactivity for p-ERK1/2 was exhibited in the pyramidal-shaped neurons and in the processes of neurons in the CA1, CA2, and CA3 regions of the ipsilateral ventral hippocampus of the control (Figure 6 A-D) and PSNL groups (Figure 6 E-H). Student’s t tests analysis revealed that the OD level for p-ERK1/2 immunostaining at the different regions of the hippocampus in the PSNL group was less than that in the control group (Figure 6I, CA1: 32.59 ± 2.05 vs. 44.80 ± 1.82, t = 4.452, p < 0.01; CA2: 19.31 ± 0.79 vs. 29.12 ± 1.83, t = 4.915 p < 0.01; CA3: 18.80 ± 1.38 vs. 23.95 ± 0.99, t = 3.031, p < 0.05, n = 4 for each group) at 28 days after ligation.

### D-serine reduces the PSNL-induced anxiety-like behavior but not nociceptive pain

**Figure 7:**
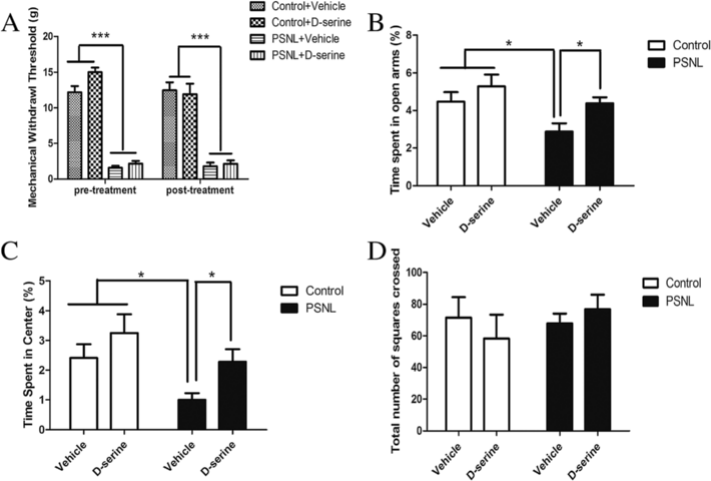
**Effect of D-serine on mechanical nociceptive threshold and anxiety-like behavior at 28 days after PSNL. Ninety minutes after D-serine treatment, MWT (A), EPM (B), and OFT (C and D) were examined.** The decreased MWT induced by PSNL was not attenuated by D-serine treatment **(A)**. The percentage of time spent in the open arm as detected by the EPM test among the different groups **(B)**. The percentage of time spent in the central zone as detected by the OFT among the different groups **(C)**. The total number of squares crossed in the open field **(D)**. *P <0.05 PSNL + Vehicle compared with Control + Vehicle, Control + D-serine and PSNL + D-serine groups. ***p < 0.001 PSNL + Vehicle and PSNL+ D-serine compared with Control + Vehicle and Control + D-serine groups.

To further determine the role of NMDAs in the neuropathic pain-related behavior induced by PSNL, D-serine was administered intraperitoneally at postoperative day 28. After 90 min of injection, animal behaviors were examined. As shown in Figure 7A, nerve injury affected the values of MWT (F_(3,25)_ = 79.96, p < 0.001), however, there was no main effect of drug treatment (F_(1,25)_ =1.376, p > 0.05) and their interaction (F_(3,25)_ =2.331, p > 0.05) on MWT. Two-way ANOVA indicated nerve injury (F_(1, 23)_ = 7.450, p < 0.05), and drug treatment (F_(1,23)_ = 5.997, p < 0.05) all affected the percentage of the time spent in the open arm detected by the EPM test. However, no main effect of their interaction (F_(1,23)_ =3.348, p > 0.05) was observed on the percentage of the time spent in the open arm (Figure 7B). Bonferroni post-tests showed that the time spent in the open arm by the PSNL group after vehicle treatment was less than that by other groups (p < 0.05). Two-way ANOVA indicated nerve injury (F_(1,23)_ = 7.873, p < 0.05), and drug treatment (F_(1,23)_ = 4.831, p < 0.05) affected the time in the central zone detected by the OFT. However, no main effect of their interaction (F_(1,23)_ = 0.8951, p > 0.05) was observed on the time in the central zone (Figure 7C). Bonferroni post-tests showed that the time spent in the central zone by the PSNL group after vehicle treatment was less than that by other groups (p < 0.05). There was no main effect of nerve injury (F_(1,25)_ = 0.3706, p > 0.05) and drug treatment (F_(1,25)_ = 0.02965, p > 0.05) and nor their interaction (F_(1, 25)_ = 0.8237,p > 0.05) on the total number of squares crossed (Figure 7D). These data imply that the activation of NMDAs only contributes to the expression of pain-related anxiety but not mechanical hypersensitivity induced by nerve ligation.

## Discussion

Peripheral nerve injury results in chronic neuropathic pain characterized by allodynia and/or affective aversive disturbances that are detrimental to patients’ well being and ability to function [3,35]. In the current study, we investigated the impact of PSNL on anxiety-like and depression-like behaviors in rats and the potential underlying molecular alterations in the hippocampus at 28 days after injury, and provided a possible explanation for behaviors related to chronic pain. We chose the time point of 28 days because early time points might not fully assimilate the impact of chronic pain on central nervous system plasticity and the development of co-morbid conditions, such as anxiety-like behavior in rats which can take more than 10 days to develop after ligation of the lumbar 5 spinal nerve [36]. More importantly, there were no alterations in anxiety-like behavior and depression-like behavior at early time points after injury (data not shown) in the current study. The results of our study showed that the anxiety-like behavior develops after PSNL as a typical feature of the neuropathic pain model in addition to the development of mechanical hypersensitivity. OFT results showed that the time spent in the central zone was significantly reduced at 28 days after injury. Moreover, this decrease did not result from a pain-evoked reduction in locomotion as in the travel distance between the two groups did not differ (data not shown). In addition, in the EPM test, rats of the PSNL group spent significantly less time in the open arms than did control rats. These data demonstrate that the anxiety-like behavior develops after injury, as seen in models of chronic inflammatory pain [37], incisional pain [24,25] and visceral pain [26]. Unexpectedly, we failed to observe any depression-like behavior at 28 days after surgery which is inconsistent with a previous report that PSNL caused a significant depression-like behavior in mice at the 4th week after surgery [38]. This discrepancy could be partially explained by the use of different species in the two studies. Although the previous study suggested that depression-related behavior was observed 6–8 weeks after insertion of a polyethylene cuff around the main branch of the right sciatic nerve in C57BL/6 J mice [39], an approach different that used in the present study, we inferred that the discrepancies in the results may be related to differences in the experimental time period (28 days vs. 6–8 weeks), the model (PSNL vs. inserting a polyethylene cuff around the main branch of the right sciatic nerve), or the species (rat vs. C57BL/6 J mice). Nevertheless, we can not preclude that there is a possibility of the development of depression-like behavior in the longer term after PSNL.

The increases in the incidence and prevalence of nerve injury-related mood disorders highlight the need for a more comprehensive understanding of the molecular mechanisms responsible for these behavioral changes. Accumulating evidence indicates that chronic neuropathic pain remains difficult to manage and causes gross reorganization and function in both cortical and subcortical structures [40,41]. The hippocampus as part of the brain structure associated with many chronic pain conditions is involved in abnormal sensitivity to neuropathic pain [9,13]. Previous studies have shown that rats with peripheral nerve injury showed changes in network activity related to the hippocampus in addition to behavioral manifestations [42,43]. The hippocampus was chosen based on evidence that the hippocampus and other limbic regions are structures responsible for regulating the emotional component, modulating the transmission of noxious pain, and processing painful stimuli [44].

Abnormal activity of glutamatergic synapses has been regarded as a mechanism in the development of neuropathic pain [45]. A recent study showed that the phosphorylation of NR1 is significantly increased in the ipsilateral dorsal horn of PSNL rats, suggesting that NR1 phosphorylation is involved in the signals underlying neuropathy [18]. A key and novel finding of this study is that NR1 and NR2B expression levels are reduced in the bilateral hippocampi, particularly in the ventral hippocampus which is not required for anxiety-like behavior like its dorsal counterpart [46]. In contrast, the expression of NR2A was not altered in PSNL rats. In humans with bipolar disorders, the expression of NR1 and NR2A transcripts in the hippocampal tissue is reduced, but there is no change in NR2B expression [47]. An earlier report showed the NR1 transcript expression is reduced in the hippocampus of mood disorders [48]. Nevertheless, these studies point to a conclusion of abnormal expression of NMDAR in affective disorders. The significance of altered expression of NR1 and NR2B expression in the hippocampus in the neuropathic pain and related behavior induced by PSNL is not clear. Activation of NMDARs with D-serine in the rostral ACC is required for the acquisition of formalin-induced pain-related negative emotion [49]. We found that D-serine treatment exhibits anxiolytic-like activity in the OPT and EPM tests without altering locomotive activity, suggesting that NMDAR plays a role in the PSNL induced pain-related negative emotions. However, D-serine did not affect mechanical hypersensitivity after PSNL, which is similar to the result for formalin-induced pain behavior following intrathecal administration of D-serine [50].

AMPA receptors are usually co-expressed with NMDARs at mature synapses, are responsible for the initial reaction to glutamate in the synapse and play distinct roles in different neuropathic pain models [51-53]. We noticed that there was no alteration of GluR1 or GluR2 expression in the hippocampus in our model. In other studies, increased GluR1 and GluR2 levels were found in spinal cord after sciatic nerve ligation [54]. In contrast, neither GluR1 nor pGluR1 is changed in the rat spinal dorsal horn after L5 spinal nerve ligation [55]. The GluR2 and GluR3 subunits of AMPA receptor are implicated in the trigeminal nerve injury-mediated hyperalgesia [56]. One possibility for these differences in study findings could be due to the use of different models and/or different locations studied.

Increasing evidence shows that mitogen-activated protein kinases such as ERK in the ACC and dorsal horn play important roles in the generation of both acute pain such as surgical pain and visceral pain, as well as chronic pain [24,26,57-59]. Accumulating evidence indicates that the maintenance of the sensory and affective components of the pain state are differentially regulated [60,61]. As most clinical neuropathic pain is chronic, it is important to address the activation of ERK1/2 at longer time points. Persistent pain was also believed to induce the synaptic plasticity in the supraspinal areas, and deactivating or activating ERK in these regions would affect the nocifensive and affective pain [62,63]. Seo et al. indicated that supraspinal phosphorylated (p)-ERK1/2 in the hippocampus has an important role in nociception and antinociception that may be downstream pathways of NMDAR activity [64]. NMDARs can activate the ERK/CREB signaling pathway in the rACC which is believed to mediate the pain-related negative emotion [65]. Therefore, ERK was selected as a potential substrate in the interaction between pain and mood in the present study. Our study demonstrated decreased phosphorylation of ERK in the hippocampus after PSNL. These results indicate the possibility that supraspinal p-ERK1/2 could play a vital role in the pathogenesis of neuropathic pain.

## Conclusion

Peripheral nerve injury causes chronic mechanical allodynia and is accompanied by anxiety-like behavior. The expression of hippocampal glutamatergic receptors and ERK phosphorylation is altered after nerve ligation, displaying an association between NMDAR and the anxiety-like behavior. Whether the decreased phosphorylation of ERK contributes to the anxiety-like behavior after ligation warrants further studies. Further molecular and neurochemical analyses are needed to elucidate the exact mechanisms of affective dimensions of pain. Our findings shed light on the possible role of hippocampal NMDARs in the pain and anxiety-like behavior induced by PSNL. These results suggest an effective strategy for developing novel drugs targeting NMDARs in the treatment of anxiety-like disorders in neuropathic pain in clinics.
